# Carbon Fibers as a New Type of Scaffold for Midbrain Organoid Development

**DOI:** 10.3390/ijms21175959

**Published:** 2020-08-19

**Authors:** Anna Tejchman, Agnieszka Znój, Paula Chlebanowska, Aneta Frączek-Szczypta, Marcin Majka

**Affiliations:** 1 Department of Transplantation, Faculty of Medicine, Institute of Pediatrics, Jagiellonian University Medical College, Wielicka 265, 30-663 Kraków, Poland; tejchman.anna@gmail.com (A.T.); paulalota0@gmail.com (P.C.); 2 Department of Biomaterials and Composites, Faculty of Materials Science and Ceramics, AGH University of Science and Technology, Al. Mickiewicza 30, 30-059 Kraków, Poland; agnieszka.znoj.12@gmail.com (A.Z.); afraczek@agh.edu.pl (A.F.-S.)

**Keywords:** organoid, Parkinson’s disease, carbon fibers, scaffold, 3D model, NURR1, LMX1A, TH, PITX3

## Abstract

The combination of induced pluripotent stem cell (iPSC) technology and 3D cell culture creates a unique possibility for the generation of organoids that mimic human organs in in vitro cultures. The use of iPS cells in organoid cultures enables the differentiation of cells into dopaminergic neurons, also found in the human midbrain. However, long-lasting organoid cultures often cause necrosis within organoids. In this work, we present carbon fibers (CFs) for medical use as a new type of scaffold for organoid culture, comparing them to a previously tested copolymer poly-(lactic-*co*-glycolic acid) (PLGA) scaffold. We verified the physicochemical properties of CF scaffolds compared to PLGA in improving the efficiency of iPSC differentiation within organoids. The physicochemical properties of carbon scaffolds such as porosity, microstructure, or stability in the cellular environment make them a convenient material for creating in vitro organoid models. Through screening several genes expressed during the differentiation of organoids at crucial brain stages of development, we found that there is a correlation between *PITX3*, one of the key regulators of terminal differentiation, and the survival of midbrain dopaminergic (mDA) neurons and tyrosine hydroxylase (*TH*) gene expression. This makes organoids formed on carbon scaffolds an improved model containing mDA neurons convenient for studying midbrain-associated neurodegenerative diseases such as Parkinson’s disease.

## 1. Introduction

Induced pluripotent stem cells (iPSCs) have made it possible to generate human neurons, including dopaminergic neurons. This has created favorable conditions for research on the human brain, its development, and related diseases [1,2]. In combination with 3D organoid cell culture technology, it is possible to obtain structures resembling individual brain regions [3]. During the development of the neural tube, key components responsible for the proliferation and differentiation of midbrain dopaminergic (mDA) neuronal progenitors as well as mDA neuron maturation and survival are expressed [3]. The expression of LIM homeobox transcription factor, *LMX1A*, is necessary for the specification of mDA neurons into the midbrain floor plate (mFP) [4,5] and, via homologue 1 muscle segment homeobox (MSX1), to inhibit the appearance of the basal plate (BP) [6]. *LMX1A* regulates key postmitotic genes involved in neuronal differentiation and survival, namely, nuclear receptor 4a2 (*NRXA2*, also known as *NURR1*) and pituitary homeobox 3 (*PITX3*) that regulate the tyrosine hydroxylase (*TH*) gene [7,8]. Thus, those late transcription factors are responsible for the transformation of mDA progenitors, from postmitotic neuroblasts [9,10] up to mature mDA neurons that are characterized by the presence of TH, an enzyme enabling dopamine synthesis [11]. Brain development takes months in primates or several years in humans, which makes brain modeling difficult—especially in 2D systems [12,13]. This generates the need to create a multicellular organoid model that will be able to survive even very long culture times. As shoswn previously [14], the most effective way to obtain functional organoids is to increase the surface-to-volume ratio of the organoid while maintaining a sufficient tissue size to form neuroepithelial buds [14]. To achieve this and recreate tissue architecture, bio-engineered constructs called scaffolds are used. One of the main goals of using scaffolds in organoids is to overcome variations in differentiation capacity and necrosis inside growing organoids during prolonged cultures. Growing cultures on scaffolds enables the creation of 3D structures resembling the human brain, the anatomy of which is fundamentally different from those found in other species. Therefore, scaffolds can help to create technology that will enable the development of tissue-like structures that reproduce brain features [13,15,16,17]. In this work we present a method of differentiating iPS cells into the 3D midbrain structure [18] using two types of scaffolds as a model for studying midbrain development and diseases, particularly Parkinson’s disease.

## 2. Results

### 2.1. Scaffolds Characterization

**Figure 1:**
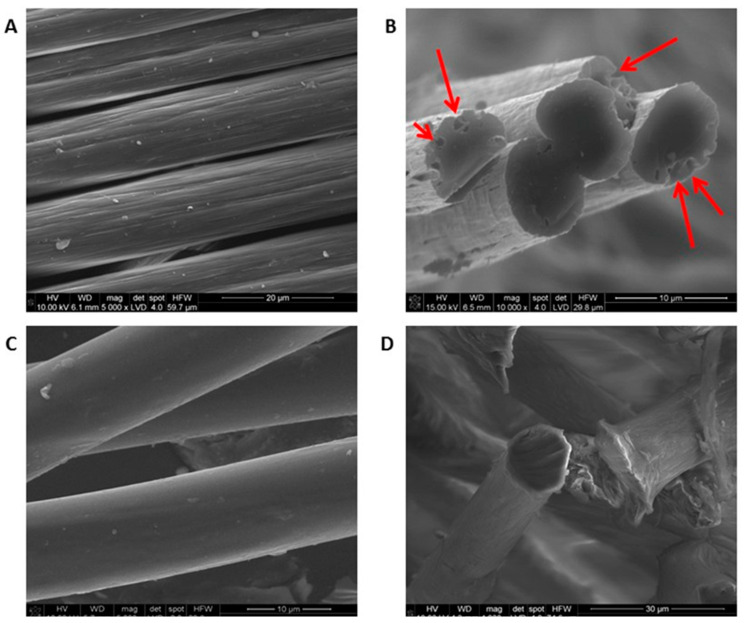
SEM microphotographs of carbon fibers (CFs) and poly (lactic-*co*-glycolic acid) (PLGA): (**A**,**C**) fiber surfaces and (**B**,**D**) their cross sections. Pores are indicated with arrows. Scale bars: 10 μm–30 μm.

**Figure 2:**
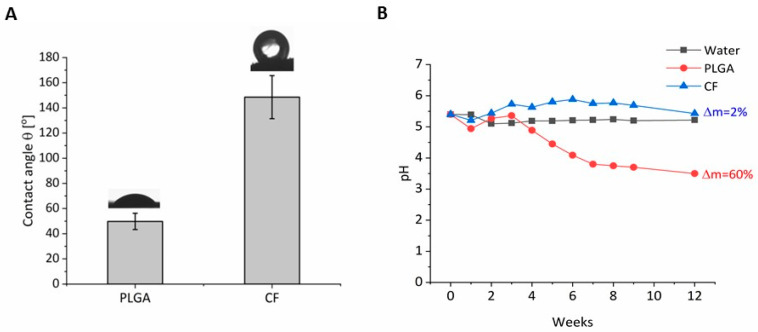
(**A**) Water contact angle for both types of fiber. The shape of the drops indicates the hydrophilic (PLGA) and hydrophobic (CF) nature of the surface of the materials. (**B**) Change in the pH of incubated fibers as a function of time.

Two types of fibrous materials differing mainly in chemical composition and morphology were proposed for this research, as follows: poly-(lactic-*co*-glycolic acid) (PLGA) fibers, the use of this material was based on reference [14]; carbon fibers (CFs) for medical purposes synthesized at the Faculty of Materials Science and Ceramics, in the Department of Biomaterials and Composites. The procedure for preparing PLGA was described in an earlier publication [19]. The morphology, microstructure, and other selected properties of the fibers were analyzed using SEM (Figure 1). The SEM micrographs illustrate the typical surface of the carbon fibers and PLGA fibers. Grooves characteristic for this material can be observed along the surface of the carbon fibers in Figure 1A. The polymer fibers (PLGA) had a smooth surface, with no irregularities (Figure 1C). Pores with a diameter of about d = 1.5 ± 0.5 μm are visible superficial to the surface of the fibers on the cross section of the CFs in Figure 1B. Such large pores were not observed in the PLGA fibers (Figure 1D). Table 1 shows the pore sizes in both types of fiber, determined using mercury porosimetry. This method makes it possible to analyze the pore size at nanoscale. There were two maxima in the pore size distributions of the investigated fibers. CF fibers were characterized by a significantly higher concentration of small (from 4 to 15 nm) and large (from 150 nm to 750 nm) pores as compared to PLGA fibers. Figure 2A presents the results of contact angle measurements on ultra-clean water by the Ström method. Carbon fibers were characterized by significant hydrophobicity, which can be observed not only based on contact angle value, but also on the droplet picture in Figure 2A. The contact angle for this sample was very high (θ = 148.5 ± 17.1°), suggesting that CFs are superhydrophobic materials. Pure carbon fibers, without any surface modification, are almost purely based on aromatic non-polar sheets, and so interaction with extremely polar molecules such as water is very weak. In the case of PLGA fibers, the contact angle was below 90°, as evidenced by the hydrophilic character of the tested sample. The value of the contact angle for this sample was θ = 49.8 ± 6.5° (Figure 2). Poly-(glycolic acid) (PGA) is a precursor of PLGA. While both belong to the family of linear aliphatic polyesters, PGA has the simplest structure and is more hydrophilic than PLGA; therefore, it determines the hydrophilic character of the PLGA copolymer.

During several weeks of incubation, the PLGA copolymer underwent hydrolytic degradation to lactide and glycolic acids, resulting in a drop in pH (Figure 2B). The initial pH drop for the PLGA fibers was observed after 4 weeks of incubation. The slow drop in pH of this sample was observed until the end of the incubation process. No changes in pH were observed for the CF sample during the entire incubation period. The degradation process was demonstrated by the mass loss of the samples, which for the PLGA sample was 60%, and was only 2% for the CF sample.

### 2.2. Generation and Characterization of Organoids from iPSCs

**Figure 3:**
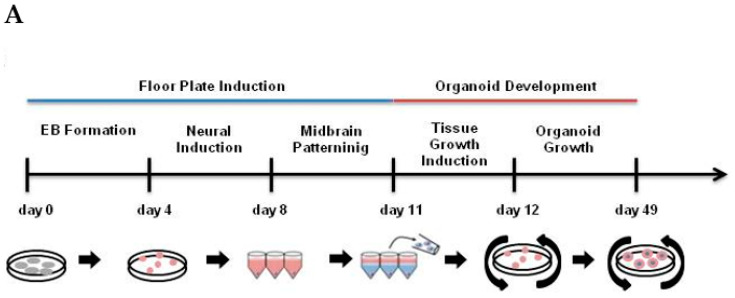

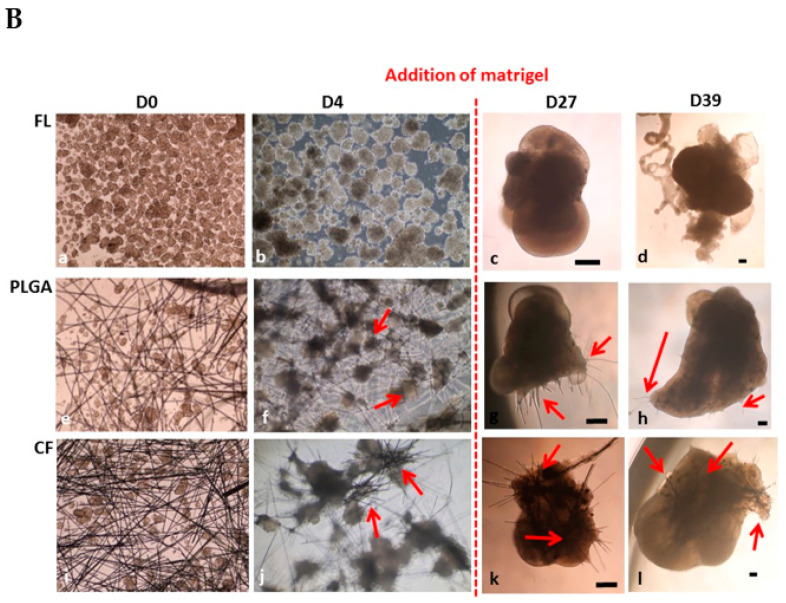
(**A**) Representation of midbrain dopaminergic (mDA)organoid formation, indicating critical steps in the procedure. Organoids were cultured without scaffold (fiber-less, FL) or on scaffolds selected from biocompatible materials (i.e., carbon fibers (CFs) and poly-(lactic-*co*-glycolic acid) (PLGA) microfilaments). The diagram shows the stages of creating organoids (Figure S1), with particular emphasis on the days when the differentiation media are changed. (**B**) Selected micrographs representing development of organoids starting from freshly detached protein-induced pluripotent stem cell (piPSC) colonies on day 0 (FL), magnification 40× (**a**); with the addition of PLGA scaffold, magnification 40× (**e**); with the addition of CF scaffold, magnification 40× (**i**). On day 4 embryoid bodies (EBs) are formed without scaffold (FL), magnification 100× (**b**); encircling the PLGA scaffold, magnification 100× (**f**); and the CF scaffold, magnification 100× (**j**). Then, a Matrigel marked with a red dotted line was added to these EBs. In the following days, the resulting organoids (**c**,**d**), PLGA scaffold organoids (**g**,**h**), and CF scaffold organoids (**k**,**l**) matured. Scaffolds incorporated into the organoid are marked with red arrows. Scale bar 200 µm.

Organoid formation began with the formation of embryoid bodies (EBs) by the dissociation of piPS cell colonies. After unsticking, piPSC colonies were transferred to non-adherent culture plates where they formed EBs for 4 days. At this stage, a scaffold obtained from PLGA or CF fibers was added to the colony (Figure 3B, Figure S1). Cell differentiation to midbrain was then performed as previously described by Jo et al. [3] (Figure 3A). In brief EBs were transferred to single V-shaped wells and grown in neural induction medium containing dual-SMAD inhibitors (Noggin and SB431542) and CHIR99021 an activator of Wnt pathway for 4 days to promote neuroectodermal differentiation. Then EBs were supplemented with midbrain patterning medium containing sonic hedgehog (SHH) and FGF8. At day 11, Matrigel was added to each well and after solidification tissue growth induction medium was added for 24 h. At day 12 organoids were transferred to non-adherent plate and media were changed for final organoid growth medium. Media were changed every 3 days and organoids were grown with orbital shaking (85 rpm). Organoids were collected and analyzed at time points critical for midbrain development: day 17 (D17), day 27 (D27), day 39 (D39), and day 49 (D49) (Figure 3).

**Figure 4:**
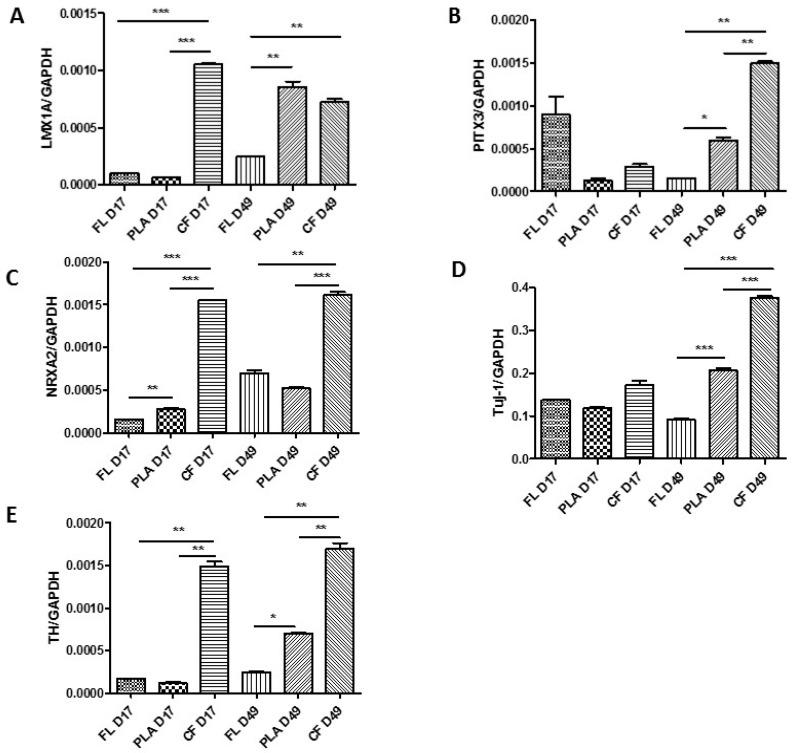
The effect of using CF and PLGA scaffolds on the expression of genes associated with the differentiation of piPSC cells into mDA neurons. The *LMX1A* (**A**), *PITX3* (**B**), *NURR1* (**C**), *TUJ1* (**D**), and TH (**E**) genes were examined. The relative expression of *LMX1A*, *PITX3*, *NURR1*(*NRXA2*), *TUJ1*, and *TH* genes was calculated by RT-qPCR using ΔCt method with *GAPDH* as constitutive control. Statistically significant differences were analyzed using one-way ANOVA with post-hoc Tukey’s multiple comparison test. piPSC FL organoids were assigned as a calibrator sample. Three independent organoids were analyzed per group (*n* = 3), the experiments were repeated two times. Results are expressed as mean values ± standard deviation (SD). * *p* < 0.05, ** *p* < 0.01, *** *p* < 0.001. *TUJ1*: beta-III-tubulin; *TH*: tyrosine hydroxylase; *NRXA2*: nuclear receptor related 1 protein; *LMX1A*: LIM homeobox transcription factor 1 alpha; *PITX3*: pituitary homeobox 3.

During the development of the organoids, the expression of crucial genes responsible for proliferation and differentiation of midbrain dopaminergic (mDA) neuron progenitors and mDA neurons were assessed (Figure 4). The expression of LIM homeobox transcription factor, *LMX1A*, was analyzed in fiber-less (FL) protein-induced pluripotent stem cells (piPSCs), piPSC PLGA, and piPSC CF organoids (Figure 4A, Figure S1). The mRNA level of *LMX1A* was highest in piPSC CF organoids on day 17, 10.08 fold higher than in piPSC FL, and 16.69 fold higher than in piPSC PLGA organoids on day 17. Its level dropped slightly in piPSC CF organoids at day 49 (0.68 fold) but increased significantly in piPSC PLGA D49 organoids in comparison to piPSC PLGA D17 organoids (13.56 fold) (Figure 4A). Two key genes involved in mDA differentiation and survival, *PITX3* and *NURR1*, were further analyzed (Figure 4B,C). *PITX3* expression level was highest on day 17 in piPSC FL organoids (Figure 4B). However, the situation was reversed at the end of the observation on day 49 when *PITX3* levels were increased significantly in the piPSC PLGA organoids (4.01 fold) relative to piPSC FL organoids and piPSC CF organoids (10.06 fold) compared to piPSC FL organoids (Figure 4B). The *NURR1* expression level was high in piPSC CF D17 organoids (9.68 fold) relative to piPSC FL D17 organoids and in piPSC CF D49 organoids (2.29 fold) compared to piPSC FL D49 organoids (Figure 4C). Its level also increased significantly in piPSC FL D49 (4.36 fold) relative to piPSC FL D17 organoids (Figure 4C). No significant changes in the expression level of *NURR1* were observed in piPSC PLGA D49 organoids (0.74 fold) relative to piPSC FL D49 organoids (Figure 4C). The expression of neuron-specific class III β-tubulin (*TUJ1*) was relatively high in all types of organoids (Figure 4D) on day 17, however, it significantly increased on day 49 in organoids growing on PLGA scaffold (2.27 fold) and even more on CF scaffold (4.12 fold) compared to the control (Figure 4D). The level of gene expression for tyrosine hydroxylase (*TH*), the characteristic marker of mDA neurons [2], was the highest in piPSC CF organoids on both day 17 and day 49, and as much as 8.75 times higher than in piPSC FL organoids on day 17 and 6.85 higher than on day 49 in piPSC FL organoids (Figure 4E). piPSC PLGA organoids also showed an increase in *TH* gene expression (2.84 fold) relative to control on day 49. *TH* expression in piPSC FL organoids increased only by 1.45 times. Interestingly, the expression level of *NURR1* in piPSC CF D17 and piPSC CF D49 did not differ significantly (1.03 fold) (Figure 4C), which correlated with the expression level for *TH* on those days (1.13 fold) (Figure 4E). Nevertheless, the expression levels of most key genes for the development of mDA neurons in the formed organoids were the highest on carbon fibers, suggesting that this scaffold is most conducive to iPS cell differentiation.

**Figure 5:**
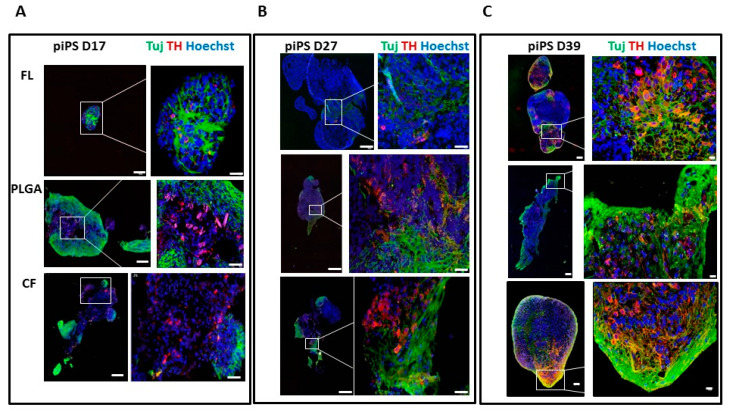
Immunofluorescent characteristics of the organoids. piPSC FL, piPSC PLGA, and piPSC CF were collected at time points corresponding to the crucial moments in the development of the brain—day 17 (D17) (**A**), day 27 (D27) (**B**), and day 39 (D39) (**C**)—fixed in 4% paraformaldehyde (PFA) then embedded in paraffin blocks and stained with neuron-specific class III β-tubulin (TUJ1), tyrosine hydroxylase (TH), the characteristic marker of mDA neurons, and nuclear marker Hoechst 33342. White scale bar 100 µm (whole organoid, left side of each panel) or 20 µm (fragment of organoid, right side of each panel).

**Figure 6:**
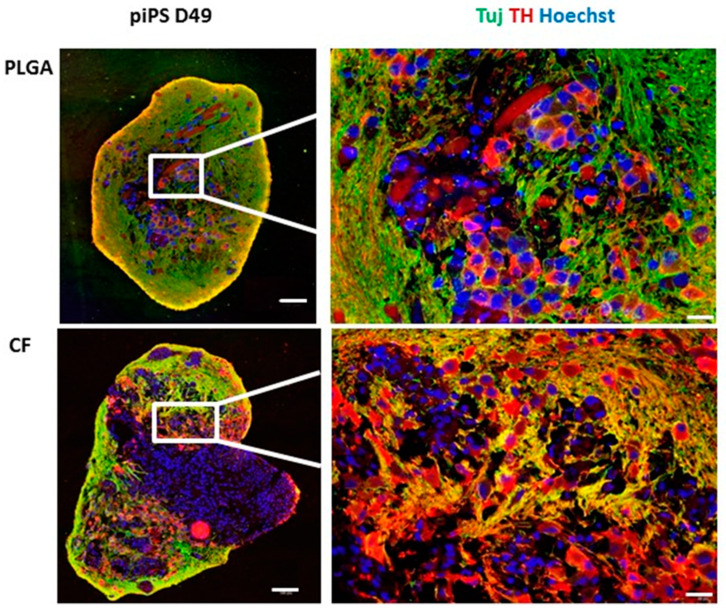
Immunofluorescent characteristics of the organoids. piPSC PLGA and piPSC CF at day 49 (D49) were collected, fixed in 4% PFA, and then embedded in paraffin blocks and stained with neuron-specific class III β-tubulin (TUJ1), tyrosine hydroxylase (TH), the characteristic marker of mDA neurons, and nuclear marker Hoechst 33342. White scale bar 100 µm (whole organoid, left side of the panel) or 20 µm (fragment of organoid, right side of the panel).

Immunohistochemistry confirmed that TH expression levels increased in the subsequent stages of differentiation, especially in the piPSC PLGA and piPSC CF organoids (Figure 5A–C and Figure 6). Interestingly, already on day 27, there was a larger number of active centers (Figure 5B) in which neurons developed, including many mDA-like neurons TUJ1+ and TH+ in the piPSC PLGA and piPSC CF organoids compared to piPSC FL. This tendency persisted in the following days of differentiation (Figure 5C and Figure 6), and the climax was reached on day 49 (Figure 6) when it was no longer possible to collect material for staining from the piPSC FL organoids due to their disintegration (Figure S2). This is related to the fact that on day 39 we began to observe progressive necrosis in the piPSC FL organoids (Figure 5C), which was not observed in any of the organoids growing on scaffolds (neither piPSC PLGA nor piPSC CF; Figure 5C), suggesting that a scaffold allows longer organoid survival and more efficient differentiation.

## 3. Discussion

Synthetic scaffolds were used to obtain the most efficient system for differentiating iPS to mDA neurons in prolonged in vitro cultures [20]. The physicochemical properties of scaffolds were examined, and the expression of key genes controlling iPS cell differentiation at various stages of development in organoids growing on these scaffolds were determined. Transcription factors that control the acquisition of mature mDA phenotype and are expressed in early post-mitotic dopaminergic neurons from E10–10.5 up to mature stages; namely, PITX3 [10] and NURR1 [9] (Figure 5) were tested at the mRNA level. NURR1 is a key factor in the survival of mDA neuroblasts and differentiation into TH+ neurons [9]. This is evidenced by studies showing that NURR1 −/− neuroblast mice are gradually lost despite acquiring the character of PITX3+ [21,22], which also indicates that NURR1 regulates *TH* expression—a gene found in mature mDA neurons. The high expression of *NURR1* in post-mitotic progenitors of mDA neurons [3,9], especially in piPSC CF organoids, indicates that the development of neuroectoderm is similar to early midbrain. The *PITX3* homeobox gene is expressed in the mDA neurons in rodents and humans [12]. It has been demonstrated that in mice, the level of *PITX3* expression correlates with TH+ mDA neurons and this in turn correlates with greater sensitivity of neurons to degeneration in Parkinson’s disease (PD) [11]. The early differentiation of mDA neurons is dependent on the *PITX3* gene, and the survival of *PITX3*-expressing mDA neurons requires high *PITX3* expression [11]. Again, PITX3 is one of the earliest mDA neuron markers [23]. PITX3 is essential for the survival of mDA SNc/A9 neurons, as evidenced by the fact that their number is significantly reduced in PITX3 −/− mice [10]. In addition, NURR1andPITX3 are essential for the survival of adult mDA neurons [11,24] and regulate each other [25,26]. In summary, NURR1 and PITX3 are some of the key regulators of terminal differentiation and of the survival of mDA neurons. This may explain why a correlation between the increasing *PITX3* and *TH* gene expression was observed in developing organoids. At the physicochemical level, microscopic analysis using the SEM technique showed the presence of grooves along CF fibers and pores, which was not observed in PLGA fibers. The presence of pores at both micro and nanoscale may contribute to better, stronger adsorption of nutrients from the culture medium and thus ensure better interaction in the cells. Higher porosity as in the case of CF also affects the growth of surface development of these materials and thus introduces more active sites capable of interacting with cells. Thus, carbon fibers are a type of porous scaffold capable of interacting with cells at both micro- and nanometric levels. Over time, the copolymer PLGA underwent hydrolytic degradation to lactide and glycolic acids, resulting in a drop in pH (Figure 2B). The initial pH drop for the PLGA fibers was observed after 4 weeks of incubation. The slow drop in pH of this sample was observed until the end of the incubation process. No changes in pH were observed for the CF sample throughout the entire incubation period. The polymer fibers are a bioresorbable material with a resorption time of 42 days. Polymeric fibers undergo hydrolytic resorption to lactide and glycolic acids, which are then eliminated from the body in metabolic cycles such as the Krebs cycle. Carbon fibers for medical purposes are a biodegradable material; they fragment into products not found naturally in the body, but their degradation products are biocompatible [27,28]. The copolymer poly(lactic-*co*-glycolic acid) (PLGA) has been developed for many years, and has been approved by the US FDA for use in drug delivery, diagnostics, and other applications of clinical and basic science research, including cardiovascular disease, cancer, vaccines, and tissue engineering [29]. Although this copolymer is widely used in medicine, there is some concern about its use in vitro due to local acidification of the environment and the possibility of negative effects on the cells. Therefore, due to the growing interest in replacement therapy in PD, cells and scaffolds that can be normalized in terms of quality, safety, and functionality before clinical use in PD are under development. Toward this end, it is necessary to obtain fully functional human mDA neurons in vitro that are capable of re-innervating the dorsal striatum and recreating the nigrostriatal pathway. To use the therapeutic potential offered by iPS cells and differentiation techniques, it is necessary to deepen the knowledge about the molecular mechanisms of this process [20]. Organoids give us an excellent model for researching brain development, improving the efficiency of differentiation, researching new drugs, and testing new therapies, including improving mDA functionality in vivo. We found that carbon fibers accelerated the growth of midbrain organoids and changed their shape in comparison to PLGA.

## 4. Materials and Methods

### 4.1. Culture of Human Induced Pluripotent Stem Cells and the Generation of Organoids

The commercially obtained protein-induced iPS (piPSC, SBI System Biosciences, Palo Alto, CA, USA) cell line was used. The piPSCs were maintained under feeder-free conditions over Matrigel-coated 6-well plates (Corning, New York, NY, USA) in DMEM/F12 media (ThermoFisher Scientific, Waltham, MA, USA) at 37 °C in a humidified CO_2_ incubator. The media was changed daily. The piPSCs line was confirmed negative for mycoplasma contamination. Organoids were formed from embryoid bodies (EBs). When cells reached confluency with tightly packed colonies, the medium was removed, and a plate was poured with dispase 1 U/mL (ThermoFisher). After a few minutes of incubation at 37 °C, dispase was gently collected, then the plate was gently rinsed with HBSS, and colonies were harvested from the plate. The cells were centrifuged and then plated on non-adherent plates (ThermoFisher) in the iPS medium without FGF. Organoids were grown in three ways: in empty medium, so-called wild type (FL), and on scaffolds (carbon fiber (CF), and surgical threads with PLGA, Polysorb 5-0 (PLGA)). Filaments were obtained from braided fibers of PLGA by mechanical dispersion as previously described by Lancaster et al., 2017 [14]. PLGA fibers were obtained from a commercial source as Vicryl sutures (Ethicon, size 5-0; Somerville, NJ, USA). Individual microfilaments were isolated from the braided fiber by mechanical shearing with a blade against a stainless-steel plate to obtain filaments of 0.5–1 mm length and 15 μm diameter. Filaments were then hydrated in embryoid body media and transferred to a 15 mL conical tube for storage at 4 °C. Filaments of carbon fibers were synthesized at the Faculty of Materials Science and Ceramics, hydrated in embryoid body media and transferred to a 15 mL conical tube for storage at 4 °C. Microfilaments were collected in a random configuration at the bottom of a low-attachment round-bottom microwell and seeded with iPS cell colonies detached with dispase to form organoid bodies. The iPS cells attached evenly along the length of PLGA microfilaments or carbon fibers, with much higher affinity to carbon fibers (Figure 3). The choice of filament material is important for the adherence of cells along the entire length. The media were changed after 2 days. Organoids were grown as described previously in [2]. Briefly, after 4 days, the detached colonies formed EBs, which were then transferred to a V-shaped plate and suspended in a medium for the induction of neurogenesis for 4 days (DMEM/F12:Neurobasal 1:1 (ThermoFisher), 1:100 N2 supplement (ThermoFisher), 1:50 B27 without vitamin A (ThermoFisher), 1% glutamine (ThermoFisher), 1% minimum essential media-nonessential amino acid (ThermoFisher), and 0.1% β-mercaptoethanol (Sigma-Aldrich, Saint Louis, MO, USA) supplemented with 1 μg/mL heparin (Sigma-Aldrich), 10 μM SB431542 (Sigma-Aldrich), 200 ng/mL Noggin (PeproTech, Inc., London, UK), 0.8 μM CHIR99021 (Sigma-Aldrich), penicillin/streptomycin 100 U/mL (ThermoFisher), and 10 μM ROCK inhibitor Y27632 (ThermoFisher)). On day 8, the medium was changed to the medium directing neurogenesis towards the midbrain for the next 3 days—100 ng/mL SHH-C25II (PeproTech) and 100 ng/mL FGF8 (PeproTech). On day 11, the media were removed from the wells and 30 μL of Matrigel was added and incubated for 30 min/37 °C. Then, the tissue-inducing medium Neurobasal medium, 1:100 N2 supplement (ThermoFisher), 1:50 B27 without vitamin A (ThermoFisher), 1% glutamine (ThermoFisher), 1% minimum essential media-nonessential amino acid (Sigma-Aldrich), and 0.1% β-mercaptoethanol (Sigma-Aldrich) supplemented with 2.5 μg/mL insulin (Sigma-Aldrich), 200 ng/mL laminin (ThermoFisher), 100 ng/mL SHH-C25II (PeproTech), and 100 ng/mL FGF8 (PeproTech) was added for 24 h. On day 12, the organoids were transferred to a non-adherent 24-well plate, where they were grown for a further 27 days (orbital shaking), and the organoid growth medium was changed every 3 days (Neurobasal medium, 1:100 N2 supplement (ThermoFisher), 1:50 B27 without vitamin A (ThermoFisher), 1% glutamine (ThermoFisher), 1% minimum essential media-nonessential amino acid (ThermoFisher), 0.1% β-mercaptoethanol (Sigma-Aldrich), 10 ng/mL BDNF (PeproTech), 10 ng/mL GDNF (PeproTech), 100 μM ascorbic acid (Sigma-Aldrich), and 125 μM db-cAMP (Sigma-Aldrich).

### 4.2. Immunohistochemistry

Organoids were collected at days 17, 27, 39, and 49 and fixed in 4% paraformaldehyde (PFA) overnight, and subsequently embedded in paraffin for sectioning. Paraffin-embedded organoids were sectioned at a thickness of 3 μm in the Pathology Laboratory of the Children Hospital in Krakow. For immunohistochemistry, organoid sections were blocked with 3% BSA and 0.5% Triton X-100 in PBS for 1 h at room temperature. The sections were incubated with primary antibodies diluted overnight and secondary antibodies for 1 h at room temperature. All sections were counterstained with Hoechst 33,342 (Sigma-Aldrich) and mounted with fluorescent mounting medium (DAKO, Glostrup, Denmark). Images were taken on an IX70 fluorescent microscope (Olympus Corporation, Tokyo, Japan). Primary antibodies were as follows: mouse anti-tubulin antibody, beta III isoform (Tuj1 MAB1637, Sigma-Aldrich), rabbit anti-tyrosine hydroxylase antibody (TH AB152, Merck-Millipore, Burlington, MA, USA). Secondary antibodies were as follows: goat anti-rabbit or anti-mouse antibodies conjugated with Alexa Fluor 555 (ThermoFisher) or Alexa Fluor 488 (ThermoFisher).

### 4.3. RNA Extraction, Reverse Transcription, Real-Time RT-PCR

Total RNAs were isolated from organoids collected at days 17, 27, 39, and 49 using RL Reagent (Eurx, Gdansk, Poland). RNA (1000 ng) was reverse-transcribed using the hMMLV kit (Promega, Madison, WI, USA) to produce the cDNA. Quantitative RT-PCR was performed using QuantStudio Real-Time PCR system (Applied Biosystem, Waltham, MA, USA). ΔCt method was applied to normalize the expression levels of each gene to that of GAPDH. TaqMan probes (ThermoFisher) were used to measure mRNA levels.

### 4.4. Carbon Fiber Synthesis

Polyacrylonitrile (PAN) (Zoltek Co., Bridgeton, MO, USA) was used as a carbon fiber precursor. PAN fibers were spun in tow from a solution in dimethylformamide (DMF). After solidification, the fibers were dry-jet wet-spun on a laboratory spinning machine using a 500-hole spinneret of 0.08 mm diameter. The fibers were stretched in two stages: in a plasticizing bath at 70 °C and under superheated steam at 135 °C. After the solvent was rinsed out, the fibers were dried between 80 and 120 °C. Before carbonization, pure PAN fibers were stabilized in an oxidizing atmosphere by a multistage process in the temperature range 150–280 °C. The oxidized fibers were carbonized at 1000 °C in an argon atmosphere. The carbon fibers were about 1 mm in length and 9.0 ± 0.6 µm in diameter.

### 4.5. Obtaining PLGA Fibers

The commercially available surgical suture with the trade name Vicryl (Ethicon, size 5-0) made of copolymer poly-(lactic-*co*-glycolic acid) in the ratio 10:90 was used as one of the materials. Individual microfilaments were isolated from the braided fiber by mechanical shearing. The obtained filaments were about 1 mm in length and 13 ± 1.7 µm in diameter.

### 4.6. Fiber Morphology and Microstructure Characterization

The fibers’ morphology and microstructure were characterized by scanning electron microscopy (Nova NanoSEM 200, FEI, Hillsboro, OR, USA). Mercury porosimetry measurements were made to determine the pore sizes in CF and PLGA fibers. The total volume of the pores and the pore size distribution in these materials were measured with a Hg porosimeter (Carlo-Erba Sorpty 1750, Milan, Italy). The contact angles of these two fibrous samples were measured by the sessile drop method using a DSA 10Mk2 analysis system (Kruss, Germany). The contact angle was calculated by averaging the results of 10 measurements.

### 4.7. Fiber Degradation Test

The fiber degradation test was performed in distilled water at 37 °C for 12 weeks. For this purpose, 10 mg of each type of fiber was weighed and placed in separate polypropylene (PP) containers and 50 mL of water was added to each container. The mass change measurement (Δm) was determined from the following formula: Δm = (m_F_ − m_I_) 100%, where m_F_ is the mass of sample after 12 weeks of incubation, and m_I_ is the mass sample before incubation.

The pH was measured weekly for 12 weeks using a watertight pH meter (model CP-401, Elmetron Co., Zabrze, Poland)

### 4.8. Statistical Analysis

Three independent organoids were analyzed per group. One-way ANOVA with Tukey’s multiple comparison test was performed using GraphPad Prism version 8.2.1 (San Diego, CA, USA) to verify statistical significance between the compared groups at each time point. Data are presented as comparison ± standard deviation.

## 5. Conclusions

Our results suggest that organoids can be used as a tool to deepen the knowledge of the molecular mechanisms of iPS cell differentiation in mDA neurons. The use of carbon scaffolds in organoid cultures allows them to survive longer in in vitro cultures, which gives them the opportunity for long-term culture to discover PD pathogenesis but also to test drugs for longer periods of time. Thus, this organoid model may provide a reproducible in vitro system to study the human midbrain and its related diseases.

## Figures and Tables

**Table 1:** Porosimetric data of CF and PLGA fibers.

Fibers	Fraction of Small Pores, 4–15 nm (%)	Fraction of Large Pores, 150–750 nm (%)
PLGA	0.67	2.46
CF	31.24	9.15

## Supplementary Materials

Supplementary Materials can be found at https://www.mdpi.com/1422-0067/21/17/5959/s1. Figure S1: Scaffolds incorporated into organoids. Figure S2: Disintegrated organoids.

## Abbreviations

BP	basal plate
CF	carbon fiber
EB	embryoid body
FL	fiber-less
iPSC	induced pluripotent stem cell
Lmx1a	LIM homeobox transcription factor
mDA	midbrain dopaminergic neuron
mFP	midbrain floor plate (mFP)
Msx1	homologue 1 muscle segment homeobox
Nurr1	Nr4a2, nuclear receptor 4a2
PD	Parkinson’s disease
piPS	protein-induced pluripotent stem cells
Pitx3	pituitary homeobox 3 or paired-like homeodomain transcription factor 3
PLGA	copolymer poly-(lactic-*co*-glycolic acid)
SEM	scanning electron microscopy
TH	tyrosine hydroxylase
Tuj1	neuron-specific class III β-tubulin
