# Supplementary material for: Carbon Fibers as a New Type of Scaffold for Midbrain Organoid Development
Source: Int J Mol Sci. 2020 Aug 19;21(17):5959. doi: 10.3390/ijms21175959 (PMC7504539; doi:10.3390/ijms21175959)
Supplement: Supplementary file 1 [file ijms-21-05959-s001.pdf]

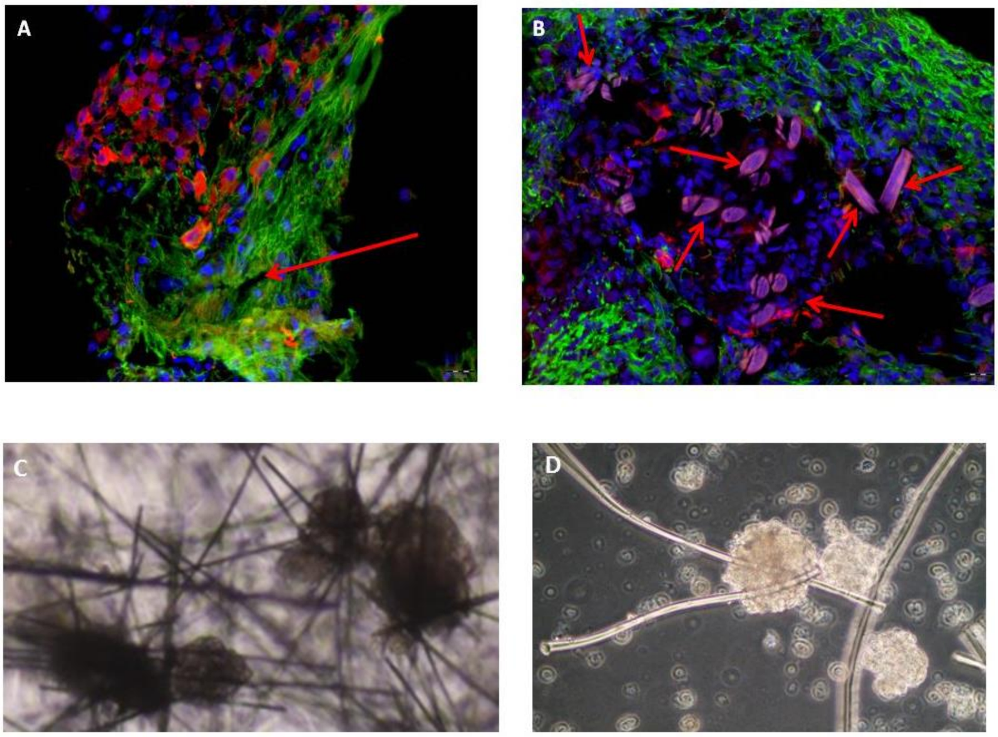

Figure 1. Scaffolds incorporated into organoids. CF scaffold A, C PLGA scaffold B, D. Scaffolds in fluorescent micrographs are indicated with arrows.

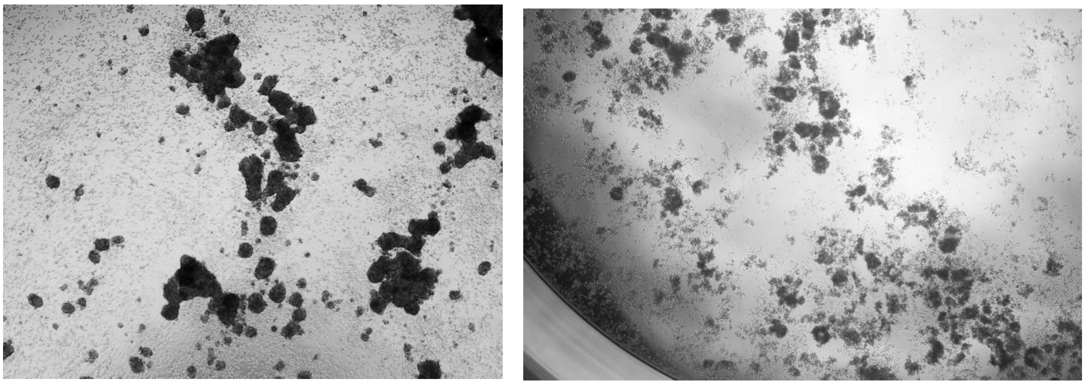

Figure 2. Disintegrated organoids.
